# N-linked glycosylation is essential for anti-tumor activities of KIAA1324 in gastric cancer

**DOI:** 10.1038/s41419-023-06083-6

**Published:** 2023-08-23

**Authors:** Rebecca Yun, Eunji Hong, Junil Kim, Bora Park, Staci Jakyong Kim, Bona Lee, Yong Sang Song, Seong-Jin Kim, Sujin Park, Jin Muk Kang

**Affiliations:** 1 GILO Institute, GILO Foundation, Seoul, 06668 Republic of Korea; 2 Interdisciplinary Program in Cancer Biology, Seoul National University, Gwanak-gu, Seoul, 08826 Republic of Korea; 3 Department of Biomedical Science, College of Life Science, Sungkyunkwan University, Suwon, Gyeonggi-do 16419 Republic of Korea; 4 School of Systems Biomedical Science, Soongsil University, Seoul, 06978 Republic of Korea; 5 WellSpan York Hospital Family Medicine Residency Program, York, PA USA; 6 International Institute for Integrative Sleep Medicine, University of Tsukuba, Tsukuba, Japan; 7 Case Western Reserve University School of Medicine, Cleveland, OH USA; 8 Department of Obstetrics and Gynecology, College of Medicine, Seoul National University, Seoul, Republic of Korea; 9 Medpacto Inc., Seoul, 06668 Republic of Korea; 10 Department of Pediatric Hematology & Oncology, University Hospitals Cleveland Medical Center, Cleveland, OH USA

**Keywords:** Gastric cancer, Glycosylation, Apoptosis, Tumour-suppressor proteins, Tumour biomarkers

## Abstract

KIAA1324 is a transmembrane protein largely reported as a tumor suppressor and favorable prognosis marker in various cancers, including gastric cancer. In this study, we report the role of N-linked glycosylation in KIAA1324 as a functional post-translational modification (PTM). Loss of N-linked glycosylation eliminated the potential of KIAA1324 to suppress cancer cell proliferation and migration. Furthermore, we demonstrated that KIAA1324 undergoes fucosylation, a modification of the N-glycan mediated by fucosyltransferase, and inhibition of fucosylation also significantly suppressed KIAA1324-induced cell growth inhibition and apoptosis of gastric cancer cells. In addition, KIAA1324-mediated apoptosis and tumor regression were inhibited by the loss of N-linked glycosylation. RNA sequencing (RNAseq) analysis revealed that genes most relevant to the apoptosis and cell cycle arrest pathways were modulated by KIAA1324 with the N-linked glycosylation, and Gene Regulatory Network (GRN) analysis suggested novel targets of KIAA1324 for anti-tumor effects in the transcription level. The N-linked glycosylation blockade decreased protein stability through rapid proteasomal degradation. The non-glycosylated mutant also showed altered localization and lost apoptotic activity that inhibits the interaction between GRP78 and caspase 7. These data demonstrate that N-linked glycosylation of KIAA1324 is essential for the suppressive role of KIAA1324 protein in gastric cancer progression and indicates that KIAA1324 may have anti-tumor effects by targeting cancer-related genes with N-linked glycosylation. In conclusion, our study suggests the PTM of KIAA1324 including N-linked glycosylation and fucosylation is a necessary factor to consider for cancer prognosis and therapy improvement.

## Introduction

Advanced technology is leading the discovery of novel cancer-related genes [1], deepening our understanding of cancer and improving therapeutic strategies. Most research on novel genes has been initially approached based on their expression level, but the functions of genes are influenced by many other factors such as interacting partners and post-transcriptional or translational modifications. To predict the action and impact of the genes on cancer precisely, altered modifications of genes or partners are being revealed through continuous research.

Post-translational modifications (PTMs) are chemical changes to a protein that alter its properties, conformation, and binding capacity [2]. PTMs control the activity, stability, and localization of proteins, thereby regulating most biological processes including cell growth, migration, and differentiation. Since PTMs are critical for the normal function of proteins, alterations are often correlated with oncogenesis, cancer development, and malignancy [3, 4].

Among PTMs, glycosylation, the attachment of glycan, is coordinated by the complex effort of nucleotide sugar transporters, glycosyltransferases, and glycosidases and initiated in the endoplasmic reticulum or Golgi apparatus [5]. Glycans added to and extending away from the surface of proteins contribute to three-dimensional conformation, overall charge, protein function, localization, and stability. N-linked glycosylation (N-glycosylation) occurs at the nitrogen atom of the asparagine (Asn) amino acid in the Asn-X-Ser/Thr consensus sequence. Alterations to N-glycosylation due to mutation and enzyme malfunction can change the biological activities of proteins, including protein trafficking and cellular signaling, leading to cancer cell growth, survival, and progression [6–8]. N-glycosylated PD-L1 was stabilized by avoiding ubiquitination and reduced binding affinity to anti-PD-L1 antibody [9, 10]. Meanwhile, deglycosylation of PD-L1 increased the detection of PD-L1 levels in cancer patients’ tissues, enhancing the prediction of anti-PD-L1/PD-1 therapeutic potential [10]. N-glycosylation of PD-1 was essential for interaction with PD-L1 and an antibody targeting N-glycosylated PD-1 showed greater efficacy in tumor regression compared to a conventional anti-PD-1 antibody [11]. VEGFR2 N-glycosylation induced interaction with galectin-1 (GAL-1), leading to tolerance to anti-VEGF treatment [12]. Thus, N-glycosylation of cancer-related proteins has been considered a crucial factor to predict cancer prognosis and therapeutic target.

Many studies have reported KIAA1324 (also known as ELAPOR1 or inceptor) as a tumor suppressor and prognostic marker in various cancers including gastric cancer [13–16]. A high KIAA1324 level was correlated with favorable prognoses and survival in cancer patients. KIAA1324 induced a strong cell growth suppression in endometrioid and nonendometrioid endometrial carcinoma through the lysosomal degradation of long-lived proteins, and its expression was accompanied by increased autophagosome and acidic vesicles [17]. In our previous study, KIAA1324 was downregulated in gastric cancer, and higher levels were associated with better patient prognoses. KIAA1324 suppressed proliferation, invasion, and drug resistance in vitro and in vivo by promoting apoptosis through its physical interaction with GRP78 (glucose-related protein 78 kDa) [13]. Furthermore, KIAA1324 has been reported to counteract insulin signaling by interacting with insulin receptor (INSR) and insulin-like growth factor 1 receptor (IGF1R) and inhibit cell proliferation, supporting the tumor-suppressive role of KIAA1324 [18]. However, although the impact of KIAA1324 in cancer has been increasing, the biological functions of KIAA1324 including PTMs have been poorly investigated.

In our current study, we identify and report the function of N-glycosylation in KIAA1324. We confirmed that N-glycosylation of KIAA1324 is essential to function as a tumor suppressor by investigating the discrepancy in the tumorigenic potential of gastric cancer cells when N-glycosylation of KIAA1324 is blocked by mutation. RNAseq also confirmed the importance of N-glycosylation and revealed many novel targets of KIAA1324 in tumor suppression. In addition, N-glycosylation was involved in the stability and localization of KIAA1324. Our findings conclude that N-glycosylation of KIAA1324 is required to inhibit gastric cancer cell progression and to induce tumor regression by inducing apoptosis and cell cycle arrest. Altogether, our data signifies that N-glycosylation of KIAA1324 is crucial to its function as a tumor suppressor and accurate prognostic indicator in gastric cancer.

## Results

### N-glycosylation is a potential PTM in KIAA1324

**Fig. 1:**
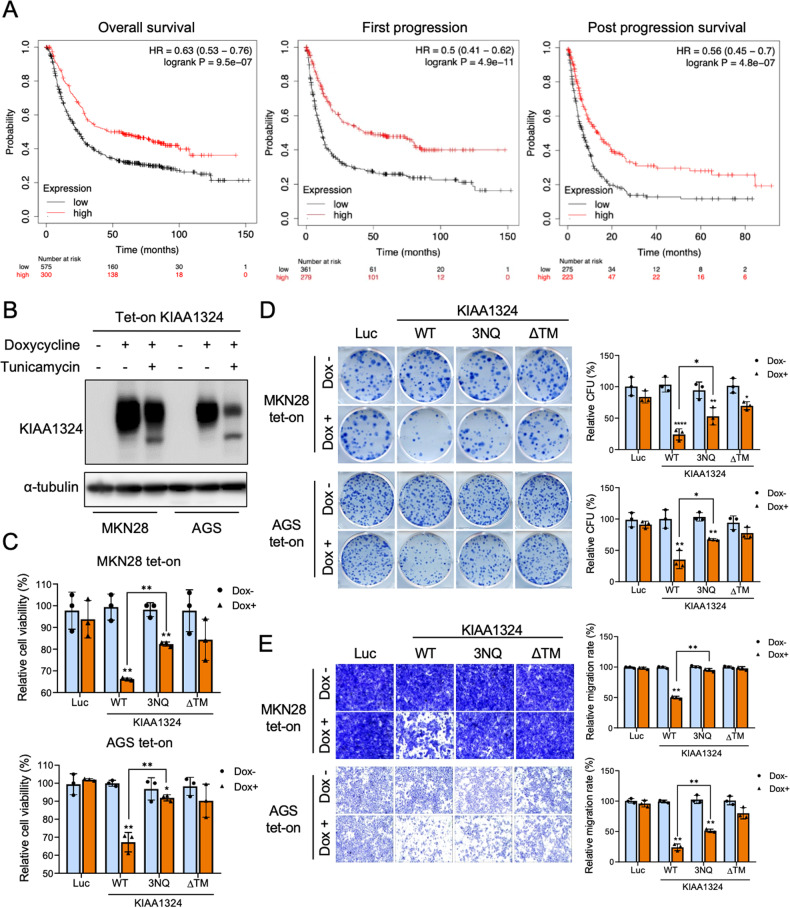
N-glycosylation of KIAA1324 is required for KIAA1324-mediated inhibition of proliferation, colony formation, and migration of gastric cancer cells. **A** Graphs of the public datasets showed an association between KIAA1324 expression level and gastric cancer patient survival. **B** Immunoblot of MKN28 and AGS cells harboring tet-on Luc measured in the presence and absence of 1 μg/mL doxycycline and 1 μg/mL tunicamycin using anti-KIAA1324. **C** Cell viability of MKN28 and AGS cells harboring tet-on Luc, KIAA1324 WT, KIAA1324 3NQ, and KIAA1324 ΔTM was measured in the presence and absence of 1 μg/mL doxycycline. Mean ± SD, *n* = 3. *P < 0.05; **P < 0.01. **D** Representative images of methylene blue–stained colonies of MKN28 and AGS cells expressing Luc, KIAA1324 WT, KIAA1324 3NQ, and KIAA1324 ΔTM. Relative colony forming unit (CFU) was calculated by dividing the colony number of doxycycline-untreated by doxycycline-treated cells. Mean ± SD, *n* = 3. *P < 0.05; **P < 0.01; ****P < 0.0001. **E** Migratory abilities of MKN28 and AGS cells expressing Luc, KIAA1324 WT, KIAA1324 3NQ, and KIAA1324 ΔTM were measured using transwell migration assay, respectively. Relative migration rates were calculated by dividing the number of doxycycline-untreated divided by doxycycline-treated cells. Mean ± SD, *n* = 3. *P < 0.05; **P < 0.01.

*KIAA1324* is one of the most significantly downregulated genes in gastric cancer tissues and cell lines. It has been established as a tumor suppressor in vitro and in vivo in gastric cancer [13]. Public datasets also demonstrate that KIAA1324 is associated with favorable survival in various cancers (Fig. 1A, Supplementary Fig. 1).

To better understand the mechanistic action of KIAA1324, we inspected the PTMs of KIAA1324 using the Uniprot database [19], which revealed N-glycosylation as a functional PTM in the KIAA1324 protein. To examine whether KIAA1324 is modified by N-glycosylation in gastric cancer cells, we treated tunicamycin, an N-glycosylation inhibitor, on KIAA1324-expressing human gastric cancer cell lines, MKN28 and AGS. By the blocking effect of N-glycosylation, the molecule size of the KIAA1324 protein was altered to a smaller form in both cell lines (Fig. 1B), indicating that N-glycosylation occurred on KIAA1324. Therefore, we investigated the specific role of N-glycosylation on KIAA1324 in gastric cancer. To confirm glycosylation and explore its importance, we generated constructs of KIAA1324 with the mutation of N-glycosylation putative sites (amino acids 153, 404, 672). Replacing asparagine residue with glutamine (N153Q, N404Q, N672Q, N153/404Q, N404/672Q, N153/672Q, and N153/404/672Q (3NQ)) inhibited N-glycosylation at the sites [20, 21] (Supplementary Fig. 2). Immunoblotting showed that KIAA1324 mutants are smaller than wild-type. The three sites mutant (3NQ) was detected as the smallest size among mutants (Supplementary Fig. 2B), suggesting that all three sites (N153/404/672) may be involved in N-glycosylation.

### N-glycosylation is required for the anti-tumor functions of KIAA1324 in cancer cells

Next, we examined the effect of N-glycosylation of KIAA1324 on in vitro tumorigenic characteristics of gastric cancer cells. We established MKN28 and AGS cell lines with a tetracycline-inducible (tet-on) viral system expressing KIAA1324 WT, 3NQ, ΔTM (deletion of the transmembrane domain), or luciferase (Luc). Our previous study showed that the loss of the transmembrane domain in KIAA1324 abolished its anti-tumor effects [13]. Therefore, this study utilized the ΔTM mutant as a nonfunctional KIAA1324 to compare with the 3NQ mutant and Luc was used as a general control.

Then, we examined whether N-glycosylation of KIAA1324 influences the tumor suppressor activity of KIAA1324 in gastric cancer cells by assessing cancerous phenotypes such as proliferation, colony formation, and migration using in vitro assays. KIAA1324 WT induction inhibited cell growth in MKN28 and AGS cells, whereas 3NQ and ΔTM mutation suppressed the KIAA1324-meditated growth inhibition (Fig. 1C). Furthermore, colony-forming activity dramatically decreased in KIAA1324 WT-expressing MKN28 and AGS cells but not in 3NQ and ΔTM-expressing cells (Fig. 1D). Moreover, in migration assay, KIAA1324 WT significantly reduced the migration of gastric cancer cells, whereas 3NQ and ΔTM mutation reduced KIAA1324-mediated inhibition of migratory activity (Fig. 1E). This demonstrated that N-glycosylation of KIAA1324 is also essential to the migratory ability of gastric cancer cells. Taken together, these results demonstrate that N-glycosylation is essential to the ability of KIAA1324 to inhibit cancer cell proliferation and migration, suggesting that N-glycosylation is crucial for KIAA1324-mediated inhibition of tumorigenic phenotypes.

### N-glycosylation is crucial for KIAA1324-mediated cancer cell apoptosis and tumor regression

**Fig. 2:**
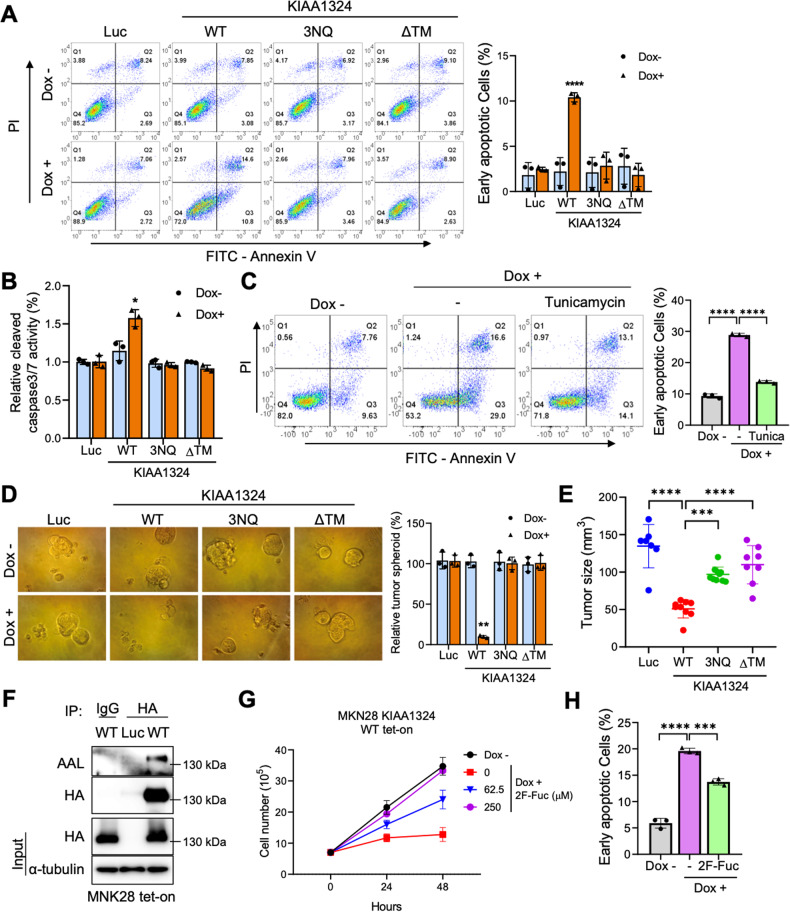
N-glycosylation is crucial for KIAA1324-mediated cancer cell apoptosis and tumor regression in vitro and in vivo. **A** Annexin V staining and flow cytometry analysis using doxycycline-induced Luc, KIAA1324 WT, KIAA1324 3NQ, and KIAA1324 ΔTM MNK28 cells with 1 μg/mL doxycycline treatment for 36 h. Annexin V-positive and PI-negative populations signify the early apoptotic cells. The quantification of the early apoptotic cell population is from a triplicate of samples graphed on the right. Mean ± SD, *n* = 3. ****P < 0.0001. **B** Cleaved caspase3/7 activity of MKN28 cells harboring tet-on Luc, KIAA1324 WT, KIAA1324 3NQ, and KIAA1324 ΔTM was measured in the presence and absence of 1 μg/mL doxycycline. Relative activity was calculated by dividing the absorbance of doxycycline-untreated by doxycycline-treated cells. Mean ± SD, *n* = 3. *P < 0.05. **C** Flow cytometry analysis of MKN28 cells harboring tet-on KIAA1324 WT treated for 36 h with 1 μg/mL doxycycline and tunicamycin. The quantification of the early apoptotic cell population from a triplicate of samples is graphed. Mean ± SD, *n* = 3. ****P < 0.0001. **D** Tumor spheroid number of MKN28 cells harboring tet-on Luc, KIAA1324 WT, KIAA1324 3NQ, and KIAA1324 ΔTM was measured in the presence and absence of 1 μg/mL doxycycline. The relative number was calculated by dividing the number of doxycycline-untreated by doxycycline-treated tumor spheroid. Mean ± SD, *n* = 3. **P < 0.01. **E** MKN28 cells harboring tet-on Luc, KIAA1324 WT, KIAA1324 3NQ, or KIAA1324 ΔTM were subcutaneously injected to each mice after 1 μg/mL doxycycline treatment. Tumor size was analyzed three weeks after injection. Mean ± SD, *n* = 8. ***P < 0.001; ****P < 0.0001. **F** Immunoprecipitation analysis of AAL in MNK28 cells expressing Luc and HA-KIAA1324 WT after 36 h treatment of 1 μg/mL doxycycline using AAL and anti-HA. **G** Proliferation of MKN28 cells harboring tet-on KIAA1324 WT measured in the presence and absence of various dosages of 2FF. Mean ± SD, *n* = 3. *P < 0.05; **P < 0.01, vs control. **H** The quantification of the early apoptotic cell population from a triplicate of samples is graphed from flow cytometry analysis of MKN28 cells harboring tet-on KIAA1324 WT treated with 1 μg/mL of doxycycline and 25uM of 2F-Fuc for 24 h. Mean ± SD, *n* = 3. ***P < 0.001; ****P < *0.0001*.

Since KIAA1324 induces apoptosis in gastric cancer cells [13], we examined whether N-glycosylation affects KIAA1324-mediated apoptosis in gastric cancer cells. We conducted annexin V staining and flow cytometry to observe apoptosis in gastric cells expressing KIAA1324 WT, 3NQ, or ΔTM in comparison to Luc (Fig. 2A). KIAA1324 WT induction increased the annexin V-positive cell population, whereas 3NQ and ΔTM did not increase the annexin V-positive cell population. Caspase activity assay showed that KIAA1324 WT expression increased cleaved caspase3/7 activities, an apoptotic marker, but 3NQ and ΔTM did not induce the activities (Fig. 2B). In addition, inhibition of N-glycosylation by tunicamycin also demolished KIAA1324-mediated apoptosis (Fig. 2C). These results indicate that N-glycosylation of KIAA1324 is required to induce apoptosis through caspase3/7 activities in gastric cancer cells.

Furthermore, to investigate whether N-glycosylation is required for KIAA1324-mediated inhibition of tumor development, we performed tumor spheroid formation assay and in vivo tumor establishment assays. Both the number and size of tumor spheroids were dramatically decreased with KIAA1324 WT induction, but not by 3NQ and KIAA1324 ΔTM induction (Fig. 2D). This data suggested that KIAA1324 inhibited tumor stemness and N-glycosylation was required. Next, to test the effect of N-glycosylation loss on in vivo tumor formation, we subcutaneously injected Luc, KIAA1324 WT, KIAA1324 3NQ, or KIAA1324 ΔTM expressing MKN28 cells into mice. Induction of KIAA1324 WT significantly inhibited the tumor growth but 3NQ and ΔTM did not reduce the tumor volume as consistent with tumor spheroid formation assay (Fig. 2E). These data indicated that N-glycosylation removal loses the inhibitory ability of tumor formation, suggesting that N-glycosylation is required for KIAA1324-induced tumor regression.

Fucosylation, a modification of N-glycoprotein mediated by fucosyltransferase [22], is involved in cancer cell adhesion, motility, and cellular signaling by regulating the functions of glycoproteins such as EGFR and TGFBR. Therefore, we investigated the possibility of fucosylation in KIAA1324. First, we detected fucosylation of KIAA1324 by immunoprecipitation assay of MNK28 cells expressing Luc or HA-tagged KIAA1324 WT and probing with *aleuria aurantia lectin* (AAL), a specific lectin containing multiple fucose-binding sites [23]. The result showed that KIAA1324 WT specifically interacted with AAL, indicating the fucosylation of KIAA1324 protein (Fig. 2F). To examine role of fucosylation in KIAA1324, we treated various dosages of a fucosyltransferase inhibitor, 2F-Peracetyl-Fucose (2F-Fuc) on MNK28 cells expressing KIAA1324 WT and performed cell proliferation assay. Inhibition of fucosylation by 2F-Fuc abolished KIAA1324-mediated cell growth inhibition in a dose-dependent manner (Fig. 2G). Subsequently, we observed that 2F-Fuc treatment decreased KIAA1324-mediated apoptosis in MKN28 cells expressing KIAA1324 WT (Fig. 2H and Supplementary Fig. 3). These results are consistent with previous reports showing that fucosylation plays a role as a tumor suppressive modification in gastric cancer [24]. Taken together, these results suggest that KIAA1324 undergoes fucosylation, and thus, has a critical impact on its functions in cell growth and apoptosis.

### RNA sequencing analysis revealed that N-glycosylation is required for KIAA1324-mediated cancer cell cycle arrest

**Fig. 3:**
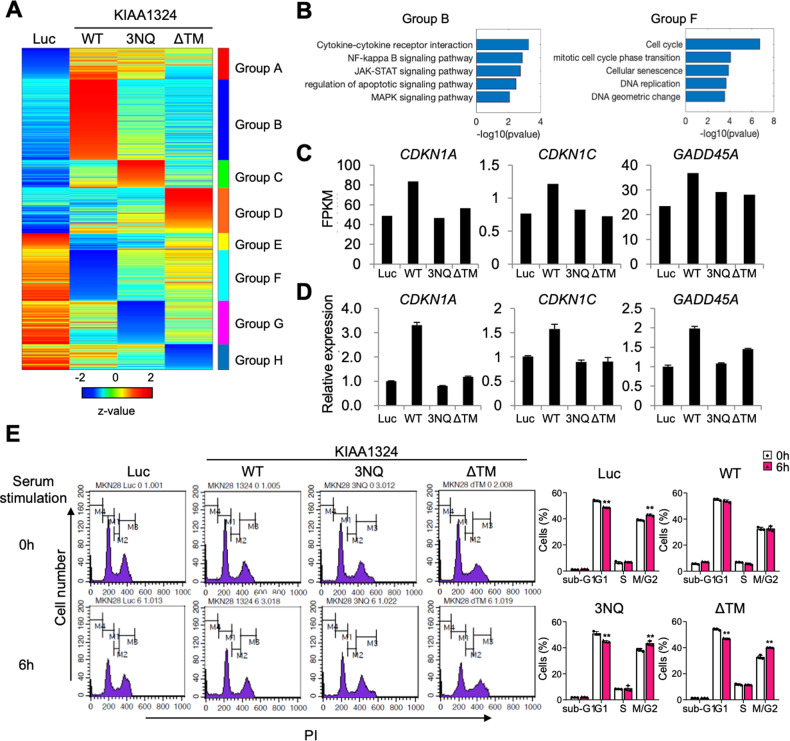
RNA sequencing analysis revealed the importance of N-glycosylation for KIAA1324-mediated cell cycle arrest. **A** Heat map of 8 DEG groups. **B** Enriched GO terms and KEGG pathways for DEG groups B and F: exclusively upregulated and downregulated in KIAA1324 WT, respectively. **C** FPKM values from RNA-sequencing and **D** relative expression levels of CDKN1A, CDKN1C, and GADD45A, genes involved in cell cycle arrest, were measured by qRT-PCR. **E** Flow cytometric analysis and graphed relative expression levels of cell cycle phase distribution upon 0 and 6 h of serum stimulation. Mean ± SD, *n* = 3. **P < 0.01.

To deepen the understanding of the role of KIAA1324 and its glycosylation in cancer, we elucidated transcriptomic changes by N-glycosylation of KIAA1324 through RNAseq from Luc, KIAA1324 WT-, 3NQ-, and ΔTM-expressing MKN28 cells (Fig. 3A).

First, to characterize the expression profiles, we performed principal component analysis (PCA) on the four RNAseq data by integrating it with our previous RNAseq data of gastric cancer cell lines and tissue [25, 26]. The expression profile of control cells (Luc) was closely located with the cancer tissue group whereas that of KIAA1324 WT-expressing cells was located with the normal tissue group, which is consistent with the tumor suppressor role of KIAA1324. The expression profiles of 3NQ and ΔTM mutant expressing cells were closer to the cancer tissue group rather than normal tissue, indicating that loss of N-glycosylation recovered KIAA1324-induced transcriptome changes (Supplementary Fig. 4).

Differentially expressed gene (DEG) analysis revealed that 3NQ and ΔTM cells expressed different gene groups compared to the WT group (Fig. 3A). Among the gene groups, we found that group F (down-regulated by KIAA1324 WT but not in 3NQ and ΔTM) was associated with the cell cycle and DNA replication pathway. Group B (up-regulated by WT but not in 3NQ and ΔTM) was associated with the regulation of apoptotic signaling pathway (Fig. 3B and Supplementary Fig. 5). Moreover, gene set enrichment analysis showed that the genes expressed in KIAA1324 WT-expressing MKN28 cells were negatively correlated with DNA replication and cell cycle pathways (Supplementary Fig. 6A). Volcano plots also confirmed eight cell cycle-related and three DNA replication-related genes were significantly (*p*-value < 0.05) down-regulated in KIAA1324 WT but not in 3NQ and ΔTM (Supplementary Fig. 6B). This gene expression profiling shows that KIAA1324 represses oncogenesis through two main pathways via N-glycosylation: cell cycle arrest and apoptosis.

In the transcriptomics analysis, cell cycle arrest genes, *CDKN1A*, *CDKN1C*, and *GADD45A*, were upregulated in KIAA1324 WT expressing cells but not in 3NQ and ΔTM expressing cells (Fig. 3C). Based on this result, we confirmed the regulation of the cell cycle-related genes by N-glycosylation of KIAA1324 using quantitative real-time polymerase chain reaction (qRT-PCR) (Fig. 3D). The KEGG pathway analysis further corroborated these results showing that most cell cycle promoting genes were downregulated and cell cycle arresting genes including *CDKN1A* (coding Cip1), *CDKN1C* (coding Kip2), and *GADD45A* were upregulated in MKN28 cells expressing KIAA1324 (Supplementary Fig. 7). This data indicates that N-glycosylation of KIAA1324 is necessary for transcriptional regulation of cell cycle arrest.

Next, we evaluated whether KIAA1324 arrests the cell cycle in gastric cancer cell lines through N-glycosylation. We found that the cell population in the G1 phase moved into the M/G2 phase in MNK28 expressing Luc, KIAA1324 3NQ, and KIAA1324 ΔTM (Fig. 3E). In contrast, the cell population in the G1 phase remained consistent in cells expressing KIAA1324 WT. This data indicates that KIAA1324 induced cell cycle arrest, but the cell cycle continues when the N-glycosylation of KIAA1324 is lost, suggesting that N-glycosylation is essential for KIAA1324-mediated cell cycle arrest.

### Gene regulatory network analysis identified key transcription factors for N-glycosylated KIAA1324-mediated anti-tumor activities

**Fig. 4:**
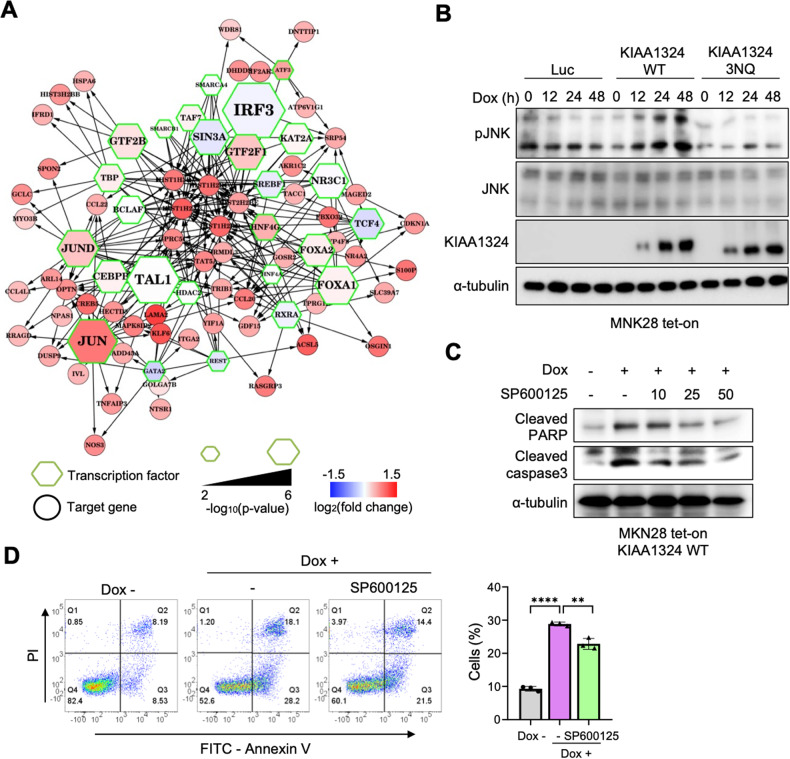
Gene regulatory network analysis identified key transcription factors and apoptosis induction through the JNK pathway mediated by N-glycosylation of KIAA1324. **A** Reconstructed gene regulatory networks for up-regulated DEGs exclusive to KIAA1324 WT, and not in 3NQ and ΔTM. **B** Immunoblot of JNK of the signaling pathway in MNK28 cells expressing KIAA1324 WT and 3NQ mutant. **C** Cleavage of caspase-3 and PARP was shown in immunoprecipitation assay performed by treating MKN28 cells harboring KIAA1324 WT with 0, 10, 25, and 50 µM of JNK inhibitor, SP600125. **D** Flow cytometry analysis of MKN28 cells harboring tet-on KIAA1324 WT treated for 36 h with 1 μg/mL doxycycline and JNK inhibitor, SP600125. The quantification of the early apoptotic cell population from a triplicate of samples is graphed. Mean ± SD, *n* = 3. **P < 0.01; ****P < 0.0001.

To identify key transcription factors for the tumor-suppressing effect of KIAA1324, we reconstructed gene regulatory networks (GRNs) with DEGs exclusive to KIAA1324 WT, and not in 3NQ and ΔTM. We reconstructed the GRNs using hypergeometric tests [27] based on the Encyclopedia of DNA element (ENCODE) and the SignaLink database [28]. The upregulated DEGs showed various enforced transcription factors’ networks including IRF3, TAL1, JUN, and FOXA (Fig. 4A), whereas the downregulated DEGs demonstrated dramatic network suppression of E2F1, a cell cycle progressor [29, 30], and NR2C2, a cancer-promoting orphan receptor [31–33] (Supplementary Fig. 8A).

Furthermore, we constructed networks with the gene group exclusively up- or down-regulated in WT using Ingenuity Pathway Analysis (IPA) to confirm these results on independent software [34]. The IPA network 1 includes many up-regulated genes and Jnk as a hub protein (Supplementary Fig. 8B). This result is consistent with the GRN analysis result since Jnk is an upstream regulator of the c-Jun transcription factor found in the GRN analysis (Fig. 4A). Since JNK is known to be an apoptosis regulator [35–37], the IPA network 1 represents that the up-regulated transcription program by N-glycosylated KIAA1324 is associated with apoptosis via JNK protein. The IPA network 2 includes many down-regulated genes and E2F1 a hub protein, which is consistent with the GRN analysis result (Supplementary Fig. 8C). This GRN and IPA network analysis strongly supports that KIAA1324 mediated tumor regression and N-glycosylation is important in its functions.

Since MAPK signaling, including p38 and JNK, were involved in apoptosis and RNAseq data showed MAPK signal pathway in upregulated DEGs by KIAA1324 WT, we examined whether KIAA1324 activates MAPKs for KIAA1324-mediated apoptosis. The phosphorylation of JNK increased with the induction of KIAA1324 WT but not 3NQ (Fig. 4B). Next, to validate the involvement of the JNK pathway in apoptosis, a JNK inhibitor SP600125 was introduced to MKN28 cells expressing KIAA1324. SP600125 decreased the level of apoptosis markers, cleaved PARP, and cleaved caspase-3, in a dose-dependent manner (Fig. 4C). In addition, apoptosis was reduced by SP600125 in gastric cells expressing KIAA1324 WT (Fig. 4D). However, when other inhibitors of MAPKs such as U0126, an Erk inhibitor, and SB203580, a p38 inhibitor were introduced, they did not reduce KIAA1324-mediated apoptosis but rather increased apoptotic cell populations. Meanwhile, tunicamycin reduced KIAA1324-mediated apoptosis (Supplementary Fig. 9). These data demonstrate that KIAA1324 induces apoptosis through the JNK pathway with N-glycosylation.

### N-glycosylation is critical for the stability and localization of KIAA1324

**Fig. 5:**
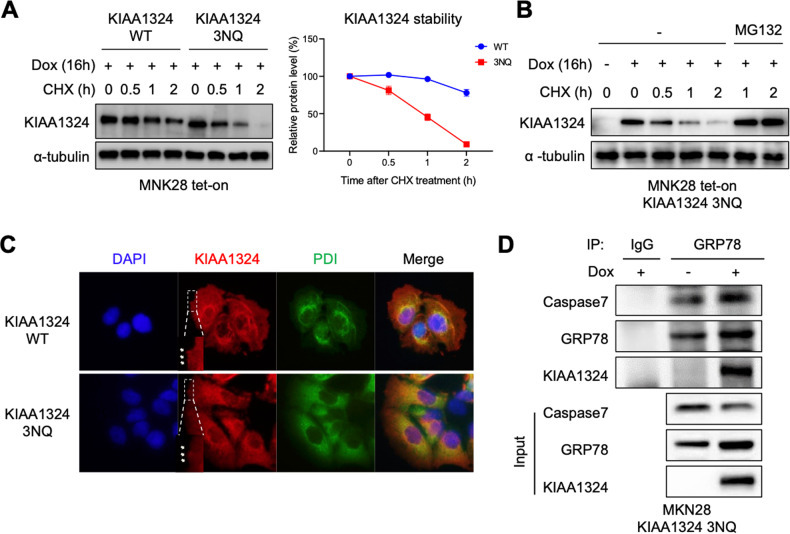
KIAA1324 glycosylation is critical for maintaining its stability, cell membrane expression, and inhibitory activity on GRP78-caspase7 interaction. **A** Protein half-life of MNK28 cells expressing KIAA1324 WT and 3NQ. Immunoblot showing KIAA1324 levels of cells treated with 1 μg/mL of doxycycline and 20 µM of CHX for indicated time. Mean ± SD, *n* = 3. *P < 0.05; **P < 0.01. **B** Immunoblot of MNK28 cells expressing KIAA1324 3NQ with CHX for the indicated time in the absence and presence of MG132. **C** Immunofluorescence assays were conducted using anti-KIAA1324, and anti-PDI antibodies in MKN28 cells harboring KIAA1324 WT and 3NQ. The nuclei were stained with DAPI. **D** Immunoprecipitation assay of interaction between GRP78 and caspase-7 was evaluated in MKN28 harboring tet-on KIAA1324 3NQ mutant after 24 h treatment of 1 μg/mL doxycycline.

Since N-glycosylation affects protein stability, localization, and binding affinity, we examined whether glycosylation of KIAA1324 is also involved in protein stability, localization, and binding affinity. To test the alteration of stability, protein levels of KIAA1324 WT and 3NQ were evaluated after we treated MNK28 expressing KIAA1324 WT and 3NQ with a protein synthesis inhibitor, cycloheximide (CHX). The half-life of the 3NQ mutant was shorter than that of WT (Fig. 5A). 3NQ mutant completely disappeared within 2 h whereas WT still maintained an 80% level. The expression of KIAA1324 3NQ mutant was restored in the presence of CHX by treatment with a proteasome inhibitor, MG132 (Fig. 5B). These data suggest that N-glycosylation of KIAA1324 is crucial for its stability by preventing it from proteasomal degradation.

Next, we examined the localization of the N-glycosylation site mutant of KIAA1324 in gastric cancer cells. Our previous study demonstrated the importance of the localization of KIAA1324 for apoptosis induction by showing that ΔTM abrogated its localization and functions. Immunofluorescence assay displayed that the cell surface level of KIAA1324 was diminished by 3NQ mutation compared with WT (Fig. 5C). This reduced membranous level of KIAA1324 3NQ might be attributed to decreased protein stability. Loss of fucosylation might also be involved in the altered cellular location of KIAA1324 as it was in HLA-DRB1 [23].

Previously, we demonstrated that KIAA1324 suppresses the antiapoptotic activity of GRP78 by interfering interaction of GRP78 with caspase-7 [13]. Therefore, we assessed whether N-glycosylation regulates the KIAA1324-mediated inhibitory effect on GRP78-caspase-7 interaction. We first examined the interaction between GRP78 and KIAA1324 mutants. Interestingly, 3NQ mutation interacted with GRP78 even stronger than WT (Supplementary Fig. 10). However, KIAA1324 3NQ induction did not affect GRP78 binding to caspase-7 despite its interaction with GRP78 (Fig. 5D). These results demonstrated that N-glycosylation is required for KIAA1324 to block the GRP78–caspase-7 interaction leading up to cell survival and antiapoptotic activity, thus inhibiting tumor formation by inducing apoptosis. Altogether, these data suggest that N-glycosylation is critical for the stability, cell surface localization, and protein regulatory activity of KIAA1324, resulting in its anti-tumor functions.

## Discussion

Most cancer-related proteins are regulated by PTMs, such as phosphorylation, SUMOylation, acetylation, ubiquitination, glycosylation, *etc*. PTMs play important roles in the protein activity involving the formation and progression of various cancers [3, 4]. PTMs largely influence and determine protein activity, intracellular distribution, stability, interaction, and life span. Understanding the phenotypes and mechanisms regulated by PTMs of cancer-associated proteins is necessary to increase targeted molecular therapy efficacy and diagnostic biomarker accuracy [38]. In this study, we demonstrated N-glycosylation is a functional PTM of KIAA1324 and revealed its importance in the biological functions and localization of KIAA1324 using in vitro and in vivo assays. RNAseq analysis identified novel targets of KIAA1324 through N-glycosylation, and cell cycle and apoptosis were confirmed as major biological processes regulated by KIAA1324. This study suggests that N-glycosylation of KIAA1324 must be considered to understand cancer development and to find a more precise diagnosis and prognosis method.

Fucosylation, a modification form of N-glycan, is crucial for cell adhesion, motility, and protein functions in cancer. Altered levels have been considered as a cancer biomarker, and targeting fucosyltransferase has been suggested as a promising cancer therapy [22]. Fucosylation enhances EGFR and TGFR signaling by regulating ligand binding affinity and cell membrane trafficking, inducing cancer cell proliferation and metastasis. Therefore, fucosylation inhibitors such as SGN-2FF have been developed for cancer therapy and are in clinical trials [39]. Meanwhile, fucosylation of HLA-DRB1 enhanced the anti-tumor effect by recruiting cytotoxic T cells in melanoma [23]. L-fucose treatment suppressed melanoma growth and enhanced anti-PD1 immunotherapy. In gastric cancer, fucosylation and Fut8, a main fucosyltransferase in cancer, were downregulated [24]. With Fut8 overexpression in gastric cancer cells, cell growth decreased. In our gastric cancer patients’ tissues, we found downregulated expression of Fut8 in cancer tissues compared to matched normal tissues in over 80% of patients (data not shown). These demonstrate that fucosylation may play a role as a tumor suppressive modification in gastric cancer. In this study, we found that KIAA1324 was fucosylated and inhibition of fucosylation blocked the anti-tumor activity of KIAA1324 (Fig. 2F, G, H). Taken together, these suggest that fucosylated KIAA1324 may be a crucial factor in fucosylation-mediated tumor suppression. Therefore, investigation of the specific role of fucosylation on KIAA1324 would be important to improve cancer therapy.

Mapping the network of DEGs by N-glycosylation of KIAA1324 implicated the regulations of various pathways through the control of transcription factors including IRF3, Jun, E2F1, and NR2C2 (Fig. 4A, Supplementary Fig. 8A). In this study, we demonstrated that JNK signaling, a well-known apoptotic signaling [35–37], was involved in KIAA1324-mediated apoptosis (Fig. 4B). However, we have yet to reveal the mechanism of KIAA1324-mediated JNK signaling regulation. In addition, although we confirmed cell cycle arrest by N-glycosylated KIAA1324, KIAA1324-mediated regulatory action in target genes was also not further investigated. Since IRF3, a tumor suppressor [40], and E2F1 [41] and NR2C2 [33], cancer promoters, have been reported to be important in cancer, investigation of the action of KIAA1324 in the regulation of these target genes is essential for understanding KIAA1324-related cancer development deeply. Identification of novel interacting partners and translocation to the functional area would reveal the mechanism of target genes’ regulation by KIAA1324 in further studies.

The role and functional structures of KIAA1324 have been characterized in various cancers and the pancreas [13–18]. In β-cells, the physical interaction of KIAA1324 with INSR-IGF1R occurs at the extracellular cysteine-rich domain. Loss of KIAA1324 leads to over-activation of INSR-IGF1R, increased β-cells proliferation, and increased glucose tolerance in vivo [18]. Since insulin/Akt signaling is strongly involved in cancer progression [42], KIAA1324-mediated regulation of insulin signaling may also suppress tumor development and invasion. In our previous study, we demonstrated that KIAA1324 binds to GRP78, an oncoprotein highly activated in various cancers, and regulates its antiapoptotic and cancer-promoting activities. When ER stress is present, GRP78 is translocated to the plasma membrane and onto the cell surface, where it promotes malignant tumorigenic properties and inhibits apoptosis signals of tumor cells through oncogenic PI3K/AKT signaling [13, 43]. In the cell membrane, KIAA1324 may regulate PI3K/AKT signaling by inhibiting GRP78 and INSR. Loss of N-glycosylation impaired the localization of KIAA1324 in the cell membrane (Fig. 5C). This might be one of the mechanisms by which the 3NQ mutation recovered cell proliferation even though the mutant bound to GRP78. Investigation of the detailed regulatory action of KIAA1324 between GRP78 and INSR would provide deeper insight into cancer progression and other diseases including diabetes.

N-glycosylation can alter the binding affinity between proteins. N-glycosylated PD-1 bound to PD-L1 strongly and signals immune suppression in T cells [11]. N-glycosylated VEGFR2 also interacted with galectin-1 and blocked the interaction between anti-VEGFR antibody and VEGFR2, leading to decreased efficacy of anti-VEGF cancer therapy [12]. On the other hand, N-glycosylation of PD-L1 inhibited GSK3β-mediated phosphorylation by avoiding interaction with GSK3β, reducing β-TrCP-induced ubiquitination, and extending half-life [9]. In this study, we found that KIAA1324 non-glycosylated mutant (3NQ) interacted with GRP78 strongly more than KIAA1323 WT (Supplementary Fig. 10). Since GRP78 controls many cancer-related proteins by physical interaction to promote cancer progression [44], we cannot exclude the possibility that GRP78 regulates KIAA1324 mutually. N-glycosylation of KIAA1324 might regulate the interaction with GRP78 and protect KIAA1324 from the control of GRP78 to induce tumor regression. In further studies, we could reveal the mutual action between GRP78 and KIAA1324.

In conclusion, our study demonstrated that the N-glycosylation of KIAA1324 is essential for the tumor suppressor to exert its antitumorigenic activities in inducing apoptosis and arresting the cell cycle via transcriptional regulation. For clinical impact, monitoring N-glycosylation of KIAA1324 would provide an efficacious method for the treatment and diagnosis of cancer. As N-glycosylation is a crucial element of tumor suppressor KIAA1324, other PTMs of KIAA1324 might also be identified and investigated in further studies.

## Material and methods

### Cell culture and transfection

Human gastric cancer cell lines, MKN28 and AGS, were acquired from the Korean Cell Line Bank. The cells were used within ten passages. All cell lines were cultured in RPMI1640 (WelGENE, Gyeongsangbuk-do, Republic of Korea, LM011-07) with 25 mmol/L HEPES (WelGENE, BB001-01), 10% FBS (WelGENE), 100 U/mL penicillin (WelGENE, LS202-02), and 100 mg/mL streptomycin (WelGENE, LS202-02). Cells were incubated at 37 °C with 5% CO2. Transfection agent FuGENE (Promega, Wisconsin, USA, E5911) was used to facilitate transfection.

### Plasmids, antibodies, and chemicals

KIAA1324 and its mutants were cloned into the pCMV-3HA vector (Clontech, California, USA) as previously described [13]. Asp (N) to Gln (Q) amino acid exchange mutation in putative N-glycosylation sites was performed by a DpnI-site directed mutagenesis method using mutagenic primers [21]. Anti-HA (Santa Cruz Biotechnology, Texas, USA, sc-7392) and anti-PARP (Santa Cruz Biotechnology, sc-8007), anti-caspase-3 (Cell Signaling Technology, Massachusetts, USA, #9662), anti-caspase-7 (Cell Signaling Technology, #9494), anti-phosphor-JNK (Cell Signaling Technology, #4668), anti-JNK (Cell Signaling Technology, #9252), anti-GRP78 (Abcam, Cambridge, United Kingdom, ab21685), anti-PDI, anti-β-actin (Abcam, ab8227), anti-KIAA1324 (Sigma, Missouri, USA, SAB2900912) and α-tubulin (Sigma, T5168) antibodies were used. 2F-Peracetyl-Fucose (Sigma, #344827), SP600125 (Sigma, S5567), SB203580 (Sigma, S8307), U0126 (Sigma, U120), tunicamycin (Sigma, T7765), and doxycycline (Sigma, #24390-14-5) chemicals were used.

### Establishment of gastric cancer cell lines expressing the *KIAA1324* gene

Stable MKN28 and AGS cell lines with doxycycline-dependent tetracycline-inducible (tet-on) KIAA1324 WT, KIAA1324 3NQ, and KIAA1324 ΔTM were established through a retroviral system. The human *KIAA1324* gene was cloned into the vector pCMV-3HA (Clontech), and the virus was produced by inserting 3HA-tagged KIAA1324 DNA into a retroviral vector, pRetroX (Clontech). MKN28 and AGS cells were infected with this virus and underwent 2 μg/mL G418 and puromycin selection.

### Cell viability assay

Cells were seeded at a density of 2 × 10^3^ cells/well for 96-well plates. To check cell viability, cells were incubated with MTT reagent (Sigma, M5655) at 37 °C and 5% CO_2_ for 4 h. After aspiration of supernatant, 100 μl DMSO was added, and the absorbance of colored solution was detected at 570 nm using SpectraMax L microplate reader (Molecular Devices, CA, USA) to quantify viability.

### Colony-forming assay

Cells were seeded at 5 × 10^3^ / well in 6-well plate density. After 10 days, the cells were fixed with 50% ethanol and stained with methylene blue. Image J software was used to quantify the cells.

### Transwell migration assay

For migration assay, 1 × 10^4^ cells were seeded in 8 μm pore membrane transwells (353097, Falcon, New York, USA). Cells were treated with 1 μg/ml doxycycline simultaneously. 24 h later, cells were stained using 0.05% Crystal violet solution (Sigma) and analyzed using Image J software.

### Tumor spheroid formation assay

1 × 10^3^ cells per well were seeded in a 96-well ultralow attachment plate (Corning, New York, USA, CLS7007). Cells were grown in RPMI 1680 supplemented with B27 (Gibco, New York, USA, 17504044), 20 ng/ml EGF (Gibco, PHG0311) and 20 ng/ml bFGF (Gibco, PHG0368), and 4 μg/ml heparin (Sigma) as described previously [45]. To induce the expression of KIAA1324, 1 μg/ml doxycycline was added. After 14 days, images were taken with a microscope and the number of spheroids with a diameter larger than 50 μm was quantified.

### Immunofluorescence Assay

Cells on the chamber slides were fixed with cold acetone at 4 °C for 5 min, blocked with 5% BSA for 1 h, and stained with DAPI, HA, or PDI antibody at 4 °C overnight. The cells were detected using Alexa Fluor 488 or 594-conjugated secondary antibody (Invitrogen). An EVOS M5000 fluorescence microscope (Thermo Fisher Scientific, Massachusetts, USA) was used to capture the immunofluorescence images.

### Immunoprecipitation and western blot analysis

Cells were harvested, washed twice with PBS, and lysed using lysis buffer with 20 mM HEPES (pH 7.5), 150 mM NaCl, 10% Glycerol, 5 mM EDTA, 1% Triton X-100, and protease inhibitor cocktail (Roche, Basel, Switzerland, 11836153001). For immunoprecipitation, the cell lysate was incubated overnight at 4 °C with indicated antibody. Dynabeads Protein G (Invitrogen, 10003D) was added and incubated for another hour. The proteins were washed four times with lysis buffer, eluted in SDS sample loading buffer, separated by SDS–polyacrylamide gel electrophoresis, transferred to polyvinylidene fluoride (PVDF) membrane (Millipore, Massachusetts, USA, IPVH00010), and blocked using 3% BSA (Millipore, C100860) for 1 h. The blots were incubated with appropriate primary antibodies overnight at 4 °C and horseradish peroxidase-conjugated secondary antibody for 1 h at room temperature. Then, they were detected using chemiluminescence (GE Healthcare Life Sciences, New Jersey, USA).

### Aleuria Aurantia Lectin detection

Western blots were were blocked using 3% BSA (Millipore, C100860) for 1 h at room temperature. They were incubated with Aleuria Aurantia Lectin (AAL), Biotinylated (Vector Laboratories, California, USA, B-1395-1) overnight at 4 °C and High Sensitivity Streptavidin-HRP (Biolegend, California, USA, 405210) for 1 h at room temperature. Then, they were detected using chemiluminescence (GE Healthcare Life Sciences, New Jersey, USA, RPN2235).

### Caspase activity assay

5 × 10^3^ cells per well were seeded in 96-well plates. After 24 h with or without doxycycline, Caspase-Glo 3/7 (Promega Corporation, Wisconsin, USA, G8090) assay was performed following the manufacturer protocol. Luminescence was detected by SpectraMax L luminescence microplate reader (Molecular Devices, California, USA).

### Annex V-positive cell population analysis

The EzWay Annexin V-FITC Apoptosis Detection Kit (KOMA Biotech, Seoul, Republic of Korea, K29100) was used according to the manufacturer protocol. Cells were trypsinized, washed twice with PBS, and stained by incubation in a binding buffer containing Annexin V-FITC and propidium iodide (PI). Then, they were analyzed using flow cytometry on the CELLQUEST program (Becton Dickinson, New Jersey, USA).

### Cell cycle analysis

To synchronize the cell cycle, cells were incubated in serum-free media for 24 h. And then, to start the cell cycle process, the media was replaced with serum-containing media. After 6 h, cells were trypsinized, washed twice with PBS buffer (pH 7.4), and fixed in cold 70% ethanol at 4 °C for 30 min. Then, they were washed twice and resuspended in PBS (with 0.1% Triton X-100, 5 μg/ml propidium iodide, and 50 μg/ml ribonuclease, A) ensuring only DNA staining. Flow cytometry analysis was conducted through the CELLQUEST program (Becton Dickinson). A total of 10,000 cells were counted.

### Mice

The in vivo experiments were performed on 6–8 weeks old BALB/c-nude female athymic nude mice of body weight 17–22 g (Orient Bio, Seongnam, Korea). The experiments were conducted in accordance with the CHA Hospital Animal Care and Use Committee standard (IACUC120021). Mice were anesthetized by isoflurane (JW Pharmaceutical Corporation, Seoul, Korea), and 1 × 10^6^ cells were injected into the flank of mice subcutaneously with matrigel (BD Sciences). Tumor sizes were measured using a vernier caliper and tumor volume was calculated using the traditional formula (sagittal dimension (mm) × (cross dimension (mm))^2^/2).

### RNA sequencing

RNA quality was assessed by analysis of rRNA band integrity on an Agilent RNA 6000 Nano kit (Agilent Technologies, California, USA). Ahead of cDNA library construction, the 1ug of total RNA and magnetic beads with Oligo (dT) were used to enrich poly (A) mRNA from it. Then, the purified mRNAs were disrupted into short fragments, and the double-stranded cDNAs were immediately synthesized. The cDNAs were subjected to end-repair, poly (A) addition, and connected with sequencing adapters using the TruSeq RNA sample prep Kit (Illumina, California, USA). The suitable fragments that were automatically purified by BluePippin 2% agarose gel cassette (Sage Science, Massachusetts, USA) were selected as templates for PCR amplification. The final library sizes and qualities were evaluated electrophoretically with an Agilent High Sensitivity DNA kit (Agilent Technologies and the fragment was found to be between 350–450 bp. Subsequently, the library was sequenced using an Illumina HiSeq2500 sequencer (Illumina).

### Transcriptome data analysis

Sequenced reads were filtered using the following criteria; reads containing more than 10% of skipped bases (marked as ‘N’), reads containing more than 40% of bases whose quality scores are less than 20 and reads whose average quality scores are less than 20. Filtered reads were mapped to the human genome GRCh37 (Ensemble release 72) using Tophat [46]. Gene expression levels were measured with Cufflinks v2.1.1 [46] using the gene annotation database of Ensembl release 72. Non-coding gene regions were removed with the mask option. To improve the accuracy of measurement, multi-read-correction, and frag-bias-correct options were applied. All other options were set to default values. DEGs were identified using Cuffdiff with default parameter setting with a significance of *p*-value < 0.05.

### Principal component analysis (PCA) with a published RNAseq data

We performed PCA on the union of the 8 DEG groups (Fig. 3A) on the new RNAseq data along with our previously published RNAseq data from the 18 gastric cancer cell lines, 18 gastric cancer tissue samples, and 16 normal gastric tissue samples [25, 26]. For the normalization between samples, we used scaled (z-transformed) log2 fragments per kilobase per million (FPKM). For the PCA, we used “pca” function implemented in MATLAB2020a.

### Gene set enrichment analysis

Enrichr [47], an enrichment analysis tool, was used to investigate the enriched GO terms and KEGG pathways for each gene group. We also run a gene-sorting-based enrichment analysis tool, Gene Set Enrichment Analysis (GSEA) [48] for cross-validation.

### Network analysis

We reconstructed the GRNs using the same strategy as our previous paper [27]. The GRN reconstruction method uses hypergeometric tests between a DEG group and a target gene group for a transcription factor (TF). The significant TFs were obtained with two criteria; *p*-value < 0.01 and the number of intersections between a DEG group and a target gene group >1. The target gene group for each TF was obtained from the Encyclopedia of DNA element (ENCODE) [49] and the SignaLink database [28]. We also used Ingenuity Pathway Analysis (IPA) [34] for the network construction.

### qRT-PCR

Cellular RNA was isolated using easy-BLUE Total RNA extraction kit (#17061, Promega) according to the manufacturer manual. A total of 1 μg of RNA and M-MLV reverse transcriptase (M1705, Promega) was used for reverse transcription. Synthesized cDNA was amplified with specific primers and TOPreal qPCR 2xPreMIX (RT500M, Enzynomics, Daejeon, Republic of Korea) and measured quantitatively using Viia 7 real-time PCR device (Applied Biosystems).

### Public data analysis

Survival benefit of various cancer patients according to KIAA1324 expression level was evaluated using a public web-based tool (Kaplan Meier plotter) [50].

### Statistical analyses

Quantitative data are presented as the mean value ± standard deviation. GraphPad Prism version 9 (GraphPad Software Inc.) was used to perform Student’s *t* tests and one-way ANOVA. Significance was determined by *P*-value: *P < 0.05; **P < 0.01.

## Supplementary information

Supplementary Figures

Reproducibility Checklist

## Data Availability

The raw data for RNA sequencing generated in this study have been deposited in the NCBI GEO database under accession code GSE239904.
