# Supplementary material for: N-linked glycosylation is essential for anti-tumor activities of KIAA1324 in gastric cancer
Source: Cell Death Dis. 2023 Aug 23;14(8):546. doi: 10.1038/s41419-023-06083-6 (PMC10447535; doi:10.1038/s41419-023-06083-6)
Supplement: Supplementary file 1 — Supplementary Figures [file 41419_2023_6083_MOESM1_ESM.docx]

**N-linked glycosylation is essential for anti-tumor activities of KIAA1324 in gastric cancer**

Rebecca Yun^1,2,#^, Eunji Hong^1,3,#^, Junil Kim^4,#^, Bora Park^5^, Staci Jakyong Kim^6^, Bona Lee^7^, Yong Sang Song^2,8^, Seong-Jin Kim^1,9^, Sujin Park^1*^, and Jin Muk Kang^10*^

^1^GILO Institute, GILO Foundation, Seoul 06668, Republic of Korea, ^2^Interdisciplinary Program in Cancer Biology, Seoul National University, Gwanak-gu, Seoul 08826, Republic of Korea, ^3^Department of Biomedical Science, College of Life Science, Sungkyunkwan University, Suwon, Gyeonggi-do 16419, Republic of Korea, ^4^School of Systems Biomedical Science, Soongsil University, Seoul 06978, Republic of Korea, ^5^WellSpan York Hospital Family Medicine Residency Program, York, Pennsylvania, USA, ^6^International Institute for Integrative Sleep Medicine, University of Tsukuba, Tsukuba, Japan, ^7^Case Western Reserve University School of Medicine, Cleveland, OH, United States, ^8^Department of Obstetrics and Gynecology, College of Medicine, Seoul National University, Seoul, Republic of Korea, ^9^Medpacto Inc., Seoul 06668, Republic of Korea, ^10^Department of Pediatric Hematology & Oncology, University Hospitals Cleveland Medical Center, Cleveland, Ohio, USA,

**Supplementary Fig. 1. KIAA1324 is a favorable prognostic marker in various cancer patients**

Graphs of the public datasets showed an association between KIAA1324 expression level and breast, lung, or ovarian cancer patients’ overall survival, relapse-free survival, or distant metastasis-free survival.

**Supplementary Fig. 2. Mutation of putative N-glycosylation sites in KIAA1324**

A) Schematic diagram of N-glycosylation loci of KIAA1324 at amino acids 153, 404, 672, asparagine (N) replaced with glutamine (Q). B) Immunoblot of KIAA1324 with one, two, or all three loci with NQ mutations.

**Supplementary Fig. 3. 2F-Fuc reduced KIAA1324-induced apoptosis**

Flow cytometry analysis of MKN28 cells harboring tet-on KIAA1324 WT treated with 1 μg/mL of doxycycline and 25uM of 2F-Fuc for 24 h.


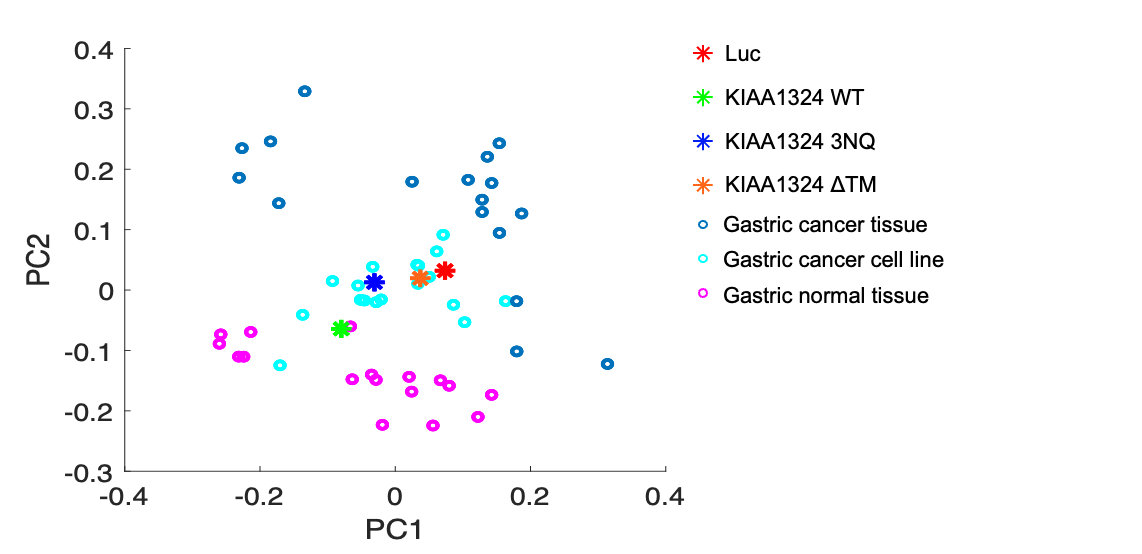


**Supplementary Fig. 4. Non-glycosylated mutation recovered KIAA1324-induced transcriptome changes**

PCA representation of RNAseq data for each Luc, KIAA1324 WT, 3NQ, and ΔTM group with a published dataset (gastric cancer cell lines, cancer tissue, and normal tissue).

**Supplementary Fig. 5.** **Enriched GO terms, and KEGG pathways for each DEG group.**

Group A are DEGs up-regulated in KIAA1324 WT and (3NQ or ΔTM). Group B are DEGs up-regulated only in WT. Group C are DEGs up-regulated only in 3NQ, Group D are DEGs up-regulated only in ΔTM. Group E are DEGs down-regulated in WT and (3NQ or ΔTM). Group F are DEGs down-regulated only in WT. Group G are DEGs down-regulated only in 3NQ, and Group H are DEGs down-regulated only in ΔTM.


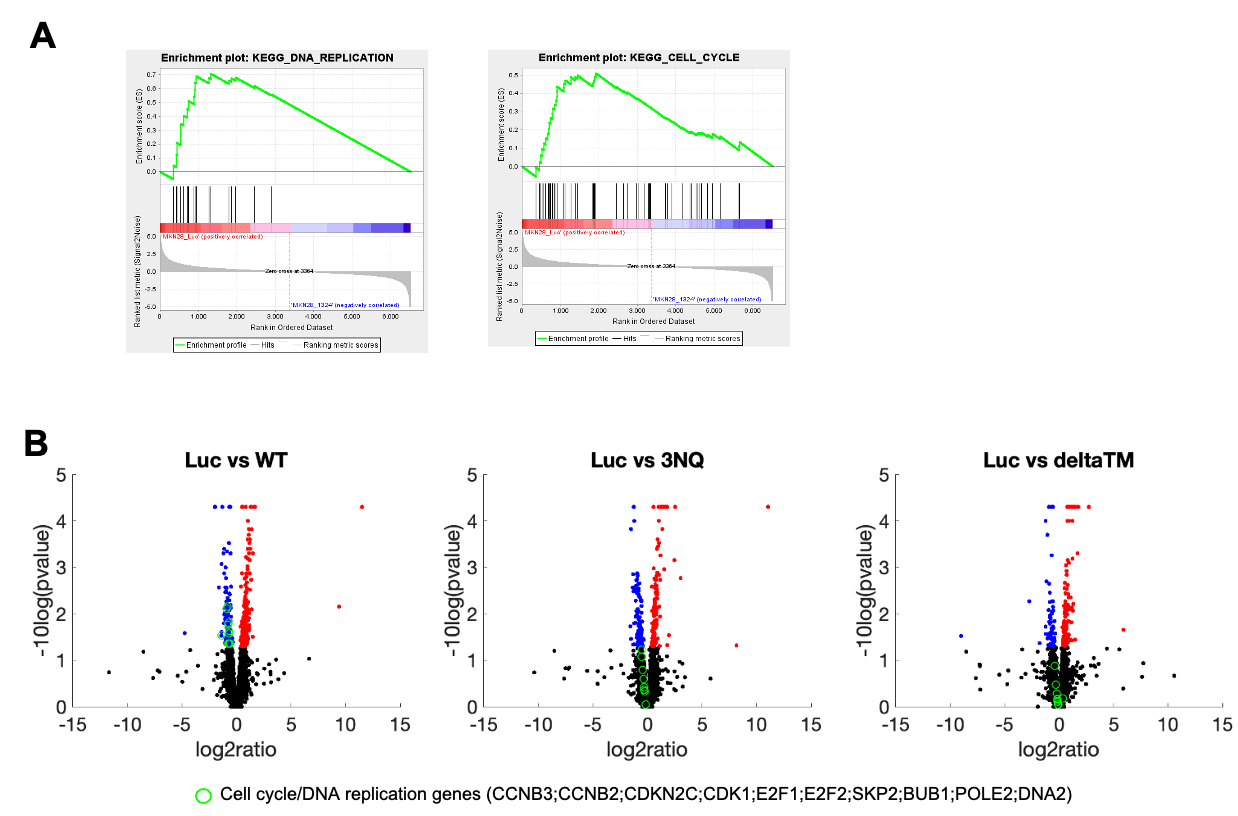


**Supplementary Fig. 6. KIAA1324 suppressed transcription of DNA replication and cell cycle-related genes**

A) Gene set enrichment analysis (GSEA) results were generated of the DNA replication and cell cycle pathway. B) Volcano plots of the DEGs in MKN28 harboring KIAA1324 WT, 3NQ, and ΔTM in comparison to negative control Luc. The blue, red, and green dots indicate the down-regulated genes, up-regulated genes, and cell cycle-related genes, respectively.

**
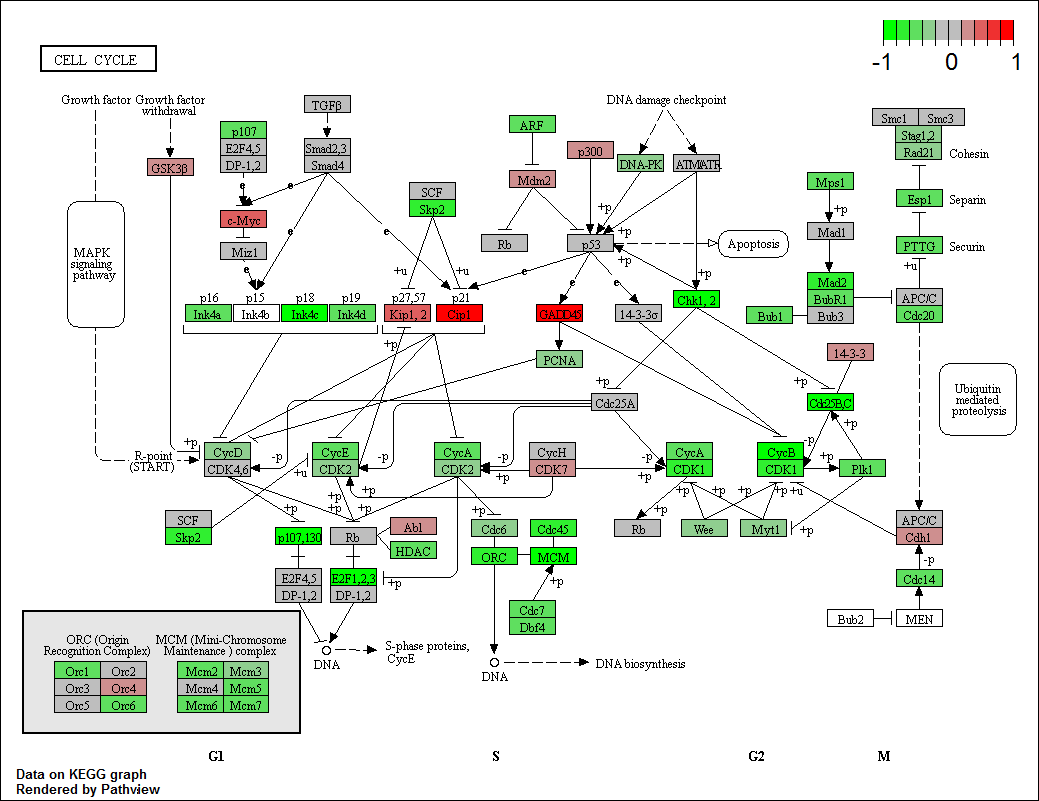
**

**Supplementary Fig. 7.** **Network of DEGs in KIAA1324 WT expressing MKN28 cells on KEGG graph rendered by Pathview.**

**Supplementary Fig. 8. Effectors regulated by N-glycosylated KIAA1324**

A) Reconstructed gene regulatory networks for down-regulated DEGs exclusive to KIAA1324 WT, and not in 3NQ and ΔTM. B) Network and regulatory effector affected by glycosylated KIAA1324-regulated genes. Ingenuity Pathway Analysis (IPA) visualized two networks with the gene group exclusively up-regulated or C) down-regulated in KIAA1324 WT not in 3NQ and ΔTM.

**Supplementary Fig. 9. Inhibition of Erk and p38 did not affect KIAA1324-mediated apoptosis**

Flow cytometry analysis of MKN28 cells harboring tet-on KIAA1324 WT treated for 36 h with 1 μg/mL doxycycline and an inhibitor: tunicamycin, U0126, or SB203580. Annexin V–positive and PI-negative populations signify early apoptotic cells. The quantification of the early apoptotic cell population from a triplicate of samples is graphed. Mean±SD.

**Supplementary Fig. 10. Loss of N-glycosylation did not block KIAA1324-GRP78 interaction**

Immunoprecipitation assay was performed in MKN28 cells harboring tet-on KIAA1324 WT, 3NQ, and ΔTM after 24 h treatment of 1 μg/mL doxycycline.

**
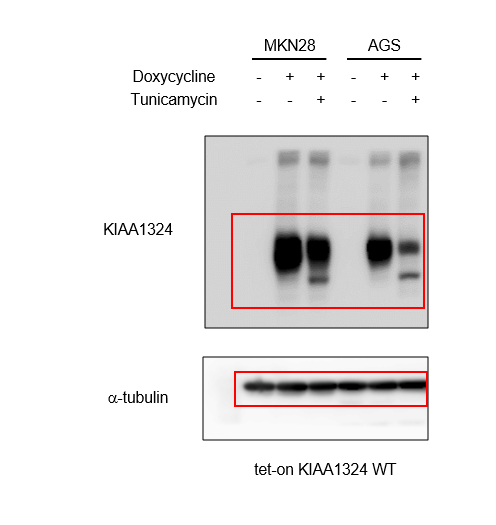
**

**Figure 1B.** **Original full length western blots**

**Figure 2F.** **Original full length western blots**

**
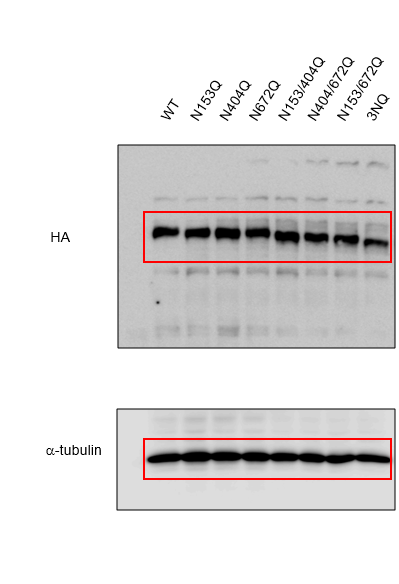
**

**Supplementary Figure 2B.** **Original full length western blots**

**
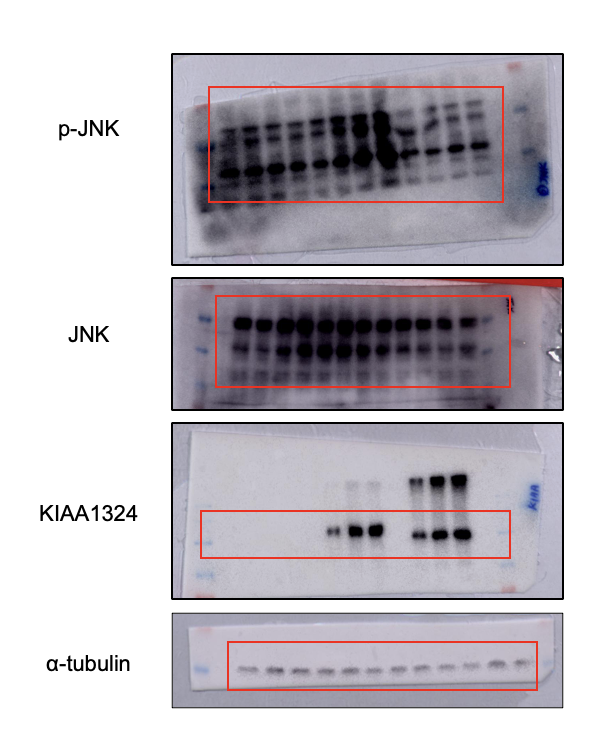
**

**Figure 4C.** **Original full length western blots**

**
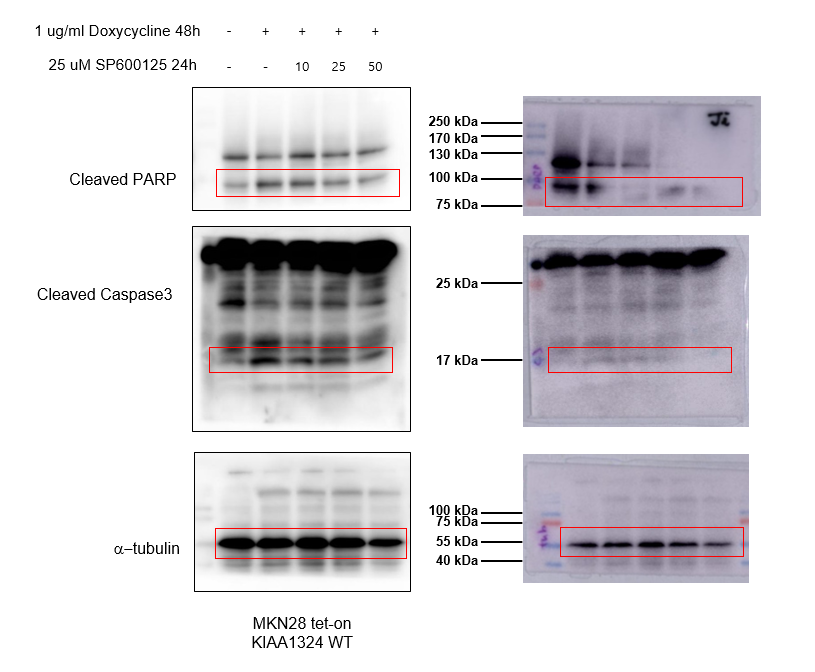
**

**Figure 4D.** **Original full length western blots**

**
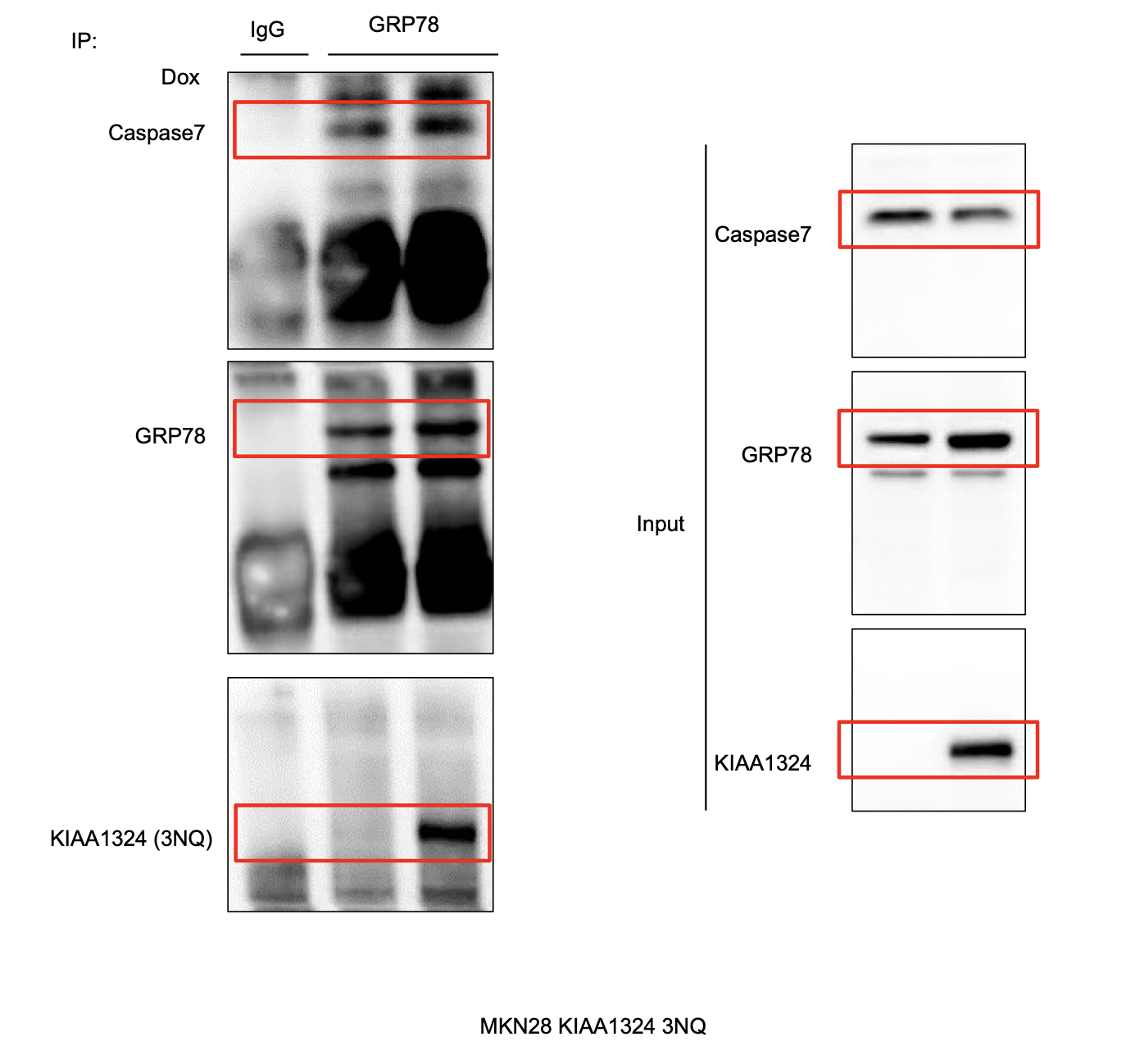
**

**Figure 4F.** **Original full length western blots**

**
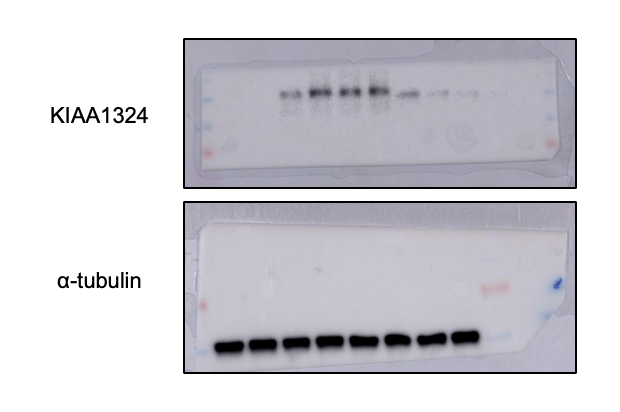
**

**Figure 5A.** **Original full length western blots**

**
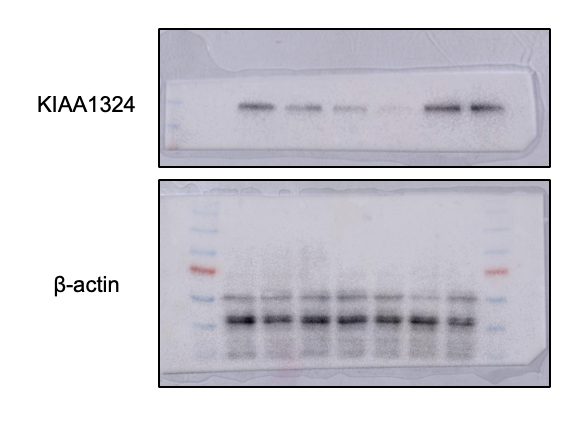
**

**Figure 5B.** **Original full length western blots**
